# An Object-Lesson for the Profession

**Published:** 1910-06-11

**Authors:** 


### An Object-Lesson for the Profession.

Whether members of the profession are in favour of nurse registration or not, if they are connected with any medical institution of importance, they must at least admit that the agitation which the nurse registrationists have engineered against the authorities of St. Bartholomew's Hospital should give every administrator pause to think. A vacancy having occurred in the matronship of St. Bartholomew's Hospital the Governors proceeded, in the ordinary way, to fill it by the appointment of the best qualified candidate they could find. The authorities threw the appointment open, and in pursuance of tradition and practice they ultimately selected a lady who was trained elsewhere than at St. Bartholomew's Hospital. We go back thirty years, which covers the period of trained nursing on a modern basis, and we find that it has been the invariable practice to appoint as a matron a lady who had been trained at some other hospital. Miss Machen was trained at St. Thomas's; her successor, Mrs. Bedford Fenwick, who was a probationer when three years' certificates were not enforced, must have obtained much of the practical knowledge which led to her appointment, when Sister Charlotte, at the London Hospital, and elsewhere; her successor, Miss Isla Stewart, was trained at St. Thomas's Hospital, and we can find no single instance where the authorities of St. Bartholomew's Hospital have appointed a matron of their own training. To state these facts is to dispose at once of any just sense of grievance on the part of the present nursing staff of St. Bartholomew's Hospital, who, we are assured, have not taken any part in the agitation created by a few ambitious individuals with axes to grind.

The moral of this agitation for the profession, however, is that, if the advanced advocates of nurse registration are to have their way, they may take every opportunity to sow discord and foster insubordination amongst the staffs of hospitals, nurse training schools, and medical charities on every occasion when the authorities, in the best interests of any such institution, may arrive at a decision which does not suit the policy of the agitators in question. Members of the medical staff of most hospitals, in the course of their experience, have learnt, that, if the patients' welfare is to be the first consideration in a hospital, and their patients are to have the best chances of recovery, it is essential that harmony shall prevail amongst the staff, and especially amongst the resident and nursing staffs and the governing body. It follows that, if we are to have nurse registration, as a matter of self-defence the members of the medical profession will be wise to examine with the utmost care the clauses of any Bill introduced into Parliament to establish it, so as to provide that the personnel of the registration board shall be freed from elements which might convert it into a mere trades union of the worst type. No one, however well-qualified otherwise, can possess strength or time enough to hold one of the higher posts in the nursing and hospital world, who actively identifies herself with politics, and consorts with those who promote, by their public action and otherwise, insubordination and disaffection within the nursing body as a whole, or in connection with a particular hospital. This principle is one which hospital authorities generally should grasp and enforce, in the best interests of the patients and the staff.

At the meeting at the Medical Society's rooms on Monday Mrs. Bedford Fenwick is reported to have said that they had decided to appeal to "their Royal President," because they had it on the authority of one of the Princesses that "George won't have any jobbery." Apart altogether from the bad taste and vulgarity of such a statement made to a crowded audience, mostly nurses, the allusion shows an ignorance of the facts, and reveals the speaker as one who cannot properly claim to voice nursing opinion. There are no more loyal subjects of His Majesty than the nurses, and they will certainly, individually and collectively, resent the offensiveness of the utterance we have ventured to quote. Is it likely that the great majority of trained nurses will permit anyone capable of such an exhibition of bad taste, aggravated as it was by the circumstances of the moment, to pose as one of their leaders for the future? The ignorance consists in the fact that King George is not the president, for St. Bartholomew's Hospital has no president at the moment, the office being vacated by the King's accession.

Altogether the attempt of the advocates of registration to enlist public sympathy by asserting that the nurses of St. Bartholomew's Hospital live in insanitary conditions and occupy a death-trap, must fail and deserves to fail. It is one more proof, of which the "insanitary conditions" referred to is another, that political women do not make satisfactory matrons. This is shown by the backward condition of the accommodation for the nursing staff, which prevailed for many years at the few hospitals where women of this type have had responsible charge. The truth is that the work of a hospital matron, especially when the hospital includes a training school, if she properly discharges her duties, is far too arduous and absorbing to leave her time to indulge in outside agitation or to take part in nursing or other politics.

